# Endothelin-1 in osteoarthritic chondrocytes triggers nitric oxide production and upregulates collagenase production

**DOI:** 10.1186/ar1489

**Published:** 2005-01-17

**Authors:** Christina Alexandra Manacu, Johanne Martel-Pelletier, Marjolaine Roy-Beaudry, Jean-Pierre Pelletier, Julio C Fernandes, Fazool S Shipkolye, Dragoslav R Mitrovic, Florina Moldovan

**Affiliations:** 1 Research Center, Sainte-Justine Hospital, Montreal, Quebec, Canada; 2 Osteoarthritis Research Unit, Centre Hospitalier de l'Université de Montréal, Hopital Notre-Dame, Montreal, Quebec, Canada; 3 Orthopaedics Research Laboratory, Department of Orthopaedics, Centre hospitalier Sacre-Coeur, Montreal, Quebec, Canada; 4 INSERM U-606, Hôpital Lariboisière, Paris, France; 5 Faculty of Dentistry, Université de Montréal, Quebec, Canada

**Keywords:** endothelin-1, metalloproteases, nitric oxide, osteoarthritis, signalling pathways

## Abstract

The mechanism of endothelin-1 (ET-1)-induced nitric oxide (NO) production, MMP-1 production and MMP-13 production was investigated in human osteoarthritis chondrocytes. The cells were isolated from human articular cartilage obtained at surgery and were cultured in the absence or presence of ET-1 with or without inhibitors of protein kinase or LY83583 (an inhibitor of soluble guanylate cyclase and of cGMP). MMP-1, MMP-13 and NO levels were then measured by ELISA and Griess reaction, respectively. Additionally, inducible nitric oxide synthase (iNOS) and phosphorylated forms of p38 mitogen-activated protein kinase, p44/42, stress-activated protein kinase/Jun-N-terminal kinase and serine-threonine Akt kinase were determined by western blot. Results show that ET-1 greatly increased MMP-1 and MMP-13 production, iNOS expression and NO release. LY83583 decreased the production of both metalloproteases below basal levels, whereas the inhibitor of p38 kinase, SB202190, suppressed ET-1-stimulated production only. Similarly, the ET-1-induced NO production was partially suppressed by the p38 kinase inhibitor and was completely suppressed by the protein kinase A kinase inhibitor KT5720 and by LY83583, suggesting the involvement of these enzymes in relevant ET-1 signalling pathways. In human osteoarthritis chondrocytes, ET-1 controls the production of MMP-1 and MMP-13. ET-1 also induces NO release via iNOS induction. ET-1 and NO should thus become important target molecules for future therapies aimed at stopping cartilage destruction.

## Introduction

Cartilage degradation in osteoarthritis (OA) and rheumatoid arthritis constitutes a major structural change in the joint, which may severely impair its function and cause pain and disability. This degradation is accompanied by the release in the synovial fluid of degraded matrix constituents that primarily result from an increased matrix catabolism [1]. Various factors are directly involved in this process. Endothelin-1 (ET-1), a potent vasoconstrictor and promitogen peptide for many cell types, including chondrocytes, was recently identified as one such factor [2,3].

ET-1 binds to the specific endothelin A or endothelin B receptors expressed on chondrocytes [4] and triggers a cascade of intracellular events, including phospholipase C activation [5], an increase in intracellular calcium [6,7], prostaglandin production [8] and nitric oxide (NO) release [9]. The effect of ET-1 on DNA and protein synthesis in chondrocytes is biphasic. The potent initial stimulatory effect of ET-1 decreases progressively with time and is followed by an inhibition [3,8]. The inhibitory effect seems to be mediated by NO and cGMP, both produced in response to ET-1 stimulation [8,9]. Additionally, we have recently demonstrated that ET-1 is significantly increased locally in OA cartilage and synovial membrane when compared with normal tissues. In OA cartilage, ET-1 is involved in cartilage catabolism through metalloprotease (MMP) regulation and the induction of type II collagen breakdown [2].

MMPs are a family of structurally related zinc-dependent neutral endopeptidases classified into subgroups of collagenases, gelatinases, stromelysins, membrane-type MMPs and other MMPs [10]. When activated, MMPs degrade a broad spectrum of substrates, including collagens and other matrix macromolecules. As a whole, MMPs play an important role in the extracellular matrix remodelling that occurs under physiological and pathological conditions. Among all the MMPs, we have recently demonstrated an induction in the synthesis, secretion and activation of two collagenases (MMP-1 and MMP-13) by ET-1 [2]. These MMPs play an active role in the progression of OA pathology as they are the most effective at initiating collagen destruction during the inflammatory process and the remodelling phase of the disease [11,12].

Another deleterious agent in joint cartilage is the NO radical [13,14], which downregulates DNA [8] and matrix synthesis [14] and upregulates matrix degradation via increased MMP synthesis [15]. Indeed, inhibition of NO production was shown to slow down the progression of OA. It has been demonstrated that, *in vitro*, NO could also upregulate MMP synthesis and activity in joint chondrocytes and cartilage [15]. *In vivo *in an OA animal model, selective inhibition of the inducible nitric oxide synthase (iNOS) provides a protective effect on OA joint tissues more specifically in regard to the degradation of the extracellular matrix and the downregulation of MMP [16].

The aim of the present study was to further investigate the role of ET-1 in human OA chondrocytes, focusing on NO, MMP-1 and MMP-13 production as well as the relevant signalling pathways activated by ET-1 in human OA chondrocytes in regard to these factors.

## Materials and methods

### Specimens

Human cartilage was obtained with the consent of 12 OA patients (mean ± standard error of the mean age, 58 ± 6 years) undergoing total knee replacement. The Institutional Ethics Committee Board of Notre Dame Hospital in Montreal, Canada approved the study protocol. Tissue specimens were embedded in paraffin, were sectioned and stained with Safranin O and fast green, and were evaluated using the Mankin histological/histochemical scale [17]. Only tissues corresponding to a moderate degree of OA severity (Mankin 3–7) were included in this study. Cartilage was sectioned from the tibial plateaus, rinsed and finely chopped, and the cells released by enzymatic digestion performed as previously described [2,11]. The cells were seeded in culture flasks at the density of 10^4 ^cells/cm^2 ^and were grown to confluence in DMEM (Gibco BRL, Burlington, ON, Canada) containing 10% heat-inactivated FCS (Hyclone, Logan, UT, USA) and 1% penicillin/streptomycin (Gibco BRL). Only first-passage-cultured cells were used.

### MMP-1 and MMP-13 quantification

MMP-1 and MMP-13 protein levels were determined in the culture media using specific ELISA assays. The ELISA assay (Amersham Biosciences Corp., Baie d'Urfé, QC, Canada) for MMP-1 specifically detected the total human MMP-1 (i.e. active MMP-1, the latent enzyme and the enzyme complexed with inhibitors such as tissue inhibitor of matrix metalloproteinases 1). The sensitivity of this assay is 1.7 ng/ml, and there is no significant cross-reactivity or interference with MMP-3, MMP-2 and MMP-9. The MMP-13 ELISA assay (R&D Systems Inc., Minneapolis, MN, USA) is a monoclonal polyclonal-based assay specific for both the active and latent MMP-13. Its sensitivity is 0.032 ng/ml, and there is no cross-reactivity with MMP-1, MMP-2, MMP-3, MMP-7, MMP-8, MMP-9 and MT1-MMP. Results are expressed as nanograms per 5 × 10^5 ^cells.

### The effect of ET-1, protein kinase inhibitors and a guanylate cyclase inhibitor (LY83583) on MMP-1, MMP-13 and NO production

MMP-1 production, MMP-13 production and NO production were studied in the absence of and in the presence of ET-1, using various inhibitors: 1 μM SB 202190 (inhibitor of p38 mitogen-activated protein [MAP] kinase), 10 μM PD 98059 (a selective mitogen-activated protein kinase kinase 1/2 [MEK1/2] inhibitor), 100 nM Wortmannin (a phosphatidyl inositol 3 kinase inhibitor), 4 μM KT5720 (a protein kinase A [PKA] inhibitor), or 2 μM LY83583 (an inhibitor of NO-dependent soluble guanylate cyclase inhibitor). All inhibitors were purchased from Calbiochem EDM Biosciences Inc. (San Diego, CA, USA), and the active concentrations chosen are based on the literature or were assayed in preliminary experiments [18,19]. ET-1 was purchased from (Sigma-Aldrich, Oakville, ON, Canada). Confluent OA chondrocytes were preincubated for 30 min with these inhibitors and then 10 nM ET-1 was added for 24 hours. Following incubation, the MMP-13 and MMP-1 protein levels and NO levels were determined in the media of six independent cultures as described in the following.

### NO determination

Nitrite (NO_2_^-^), a stable end product of NO, was measured in the media of cultured cells using a spectrophotometric method based on the Griess reaction [20]. To examine the effects of ET-1 on NO production, a dose–response curve was performed by incubating OA chondrocytes for 24 hours with increased concentrations (0–100 nM) of ET-1, or by pretreating with protein kinase inhibitors or a guanylate cyclase inhibitor and ET-1 as already described. NO production was also evaluated in the presence of the iNOS inhibitor L-NIL (L-N^6 ^(1-iminoethyl)lysine) (Calbiochem EDM Biosciences Inc.). Chondrocytes were preincubated for 30 min with 0–50 μM L-NIL and were then incubated for 24 hours with 10 nM ET-1. The media were collected and the released NO levels were determined. Results are expressed as nanomoles per 5 × 10^5 ^cells ± standard error of the mean or as a percentage of the control cultures.

### Western blot

Confluent OA chondrocytes were incubated in the presence of or in the absence (control) of 10 nM ET-1, and the cells were lysed in 0.2 ml lysis buffer (25 mM HEPES, 5 mM MgCl_2_, 1 mM EDTA, 1 mM PMSF, 10 μg/ml pepstatin, 10 μg/ml leupeptin, pH 7.5). The protein concentration of the lysate was determined with the Bradford dye assay (Bio-Rad Laboratories, Hercules, CA, USA). For western blot, 10 μg lysate protein was separated by electrophoresis on a 10% SDS discontinuous gradient polyacrylamide gel. Separated proteins were then transferred electrophoretically onto a nitrocellulose membrane (Hybond C extra; Amersham, Pharmacia Biotech, Chalfont St Giles, UK). The membranes were immersed overnight in the Super Block Blocking buffer (Pierce, Rockford, IL, USA), rinsed and incubated for 24 hours at 4°C with one of the mouse monoclonal primary antibodies (New England Biolabs, Mississauga, ON, Canada) specifically recognizing phosphorylated p38 or total p38 (dilution, 1/1000), phosphorylated p44/42 (dilution, 1/5000), phosphorylated Akt (dilution, 1/2000), phosphorylated stress-activated protein kinase/Jun-N-terminal kinase (SAP/JNK) (dilution, 1/1000), or actin C-terminal fragment (dilution, 1/5000). iNOS was detected with a rabbit polyclonal antibody (dilution, 1/1000; Santa Cruz Biotechnology Inc., Santa Cruz, CA, USA).

Following incubation with primary antibody, membranes were carefully washed and reincubated for 1 hour at 4°C with a second antibody (anti-rabbit IgG). Anti-mouse horseradish peroxidase-conjugated IgG (dilution, 1/25,000) was used for the detection of the monoclonal antibody, and sheep anti-rabbit horseradish peroxidase-conjugated IgG (dilution, 1/40,000) was used for the polyclonal antibody. Detection was performed using the Super Signal Ultra Western blot chemiluminescence system (Pierce) [11].

### Apoptosis

Apoptosis was investigated in OA chondrocytes cultured on Lab-Tec chamber slides (Nalge Nunc International, Naperville, IL, USA). At confluence, the cells were rinsed and incubated at 37°C for 72 hours in DMEM containing 2.5% heat-inactivated FCS in the absence of or in the presence of 10 nM human recombinant ET-1. Apoptotic cells were detected by *in situ *staining using the TUNEL method (Trevigen Inc., Gaithersburg, MD, USA). Both pro-apoptotic Bad and anti-apoptotic Bcl2 proteins were determined by immunocytochemical detection using specific anti-Bad and anti-Bcl2 antibodies (Upstate Biotechnology, Lake Placid, NY, USA). The results are expressed as the mean percentage of positively stained cells according to a previously published method [21,22].

### Statistical analysis

Data are expressed as the mean ± standard error of the mean of five or six independent cultures. Statistical significance was assessed by the Mann–Whitney test, and *P *< 0.05 was considered significant.

## Results

### ET-1 induces MMP-1 and MMP-13 production

**Figure 1:**
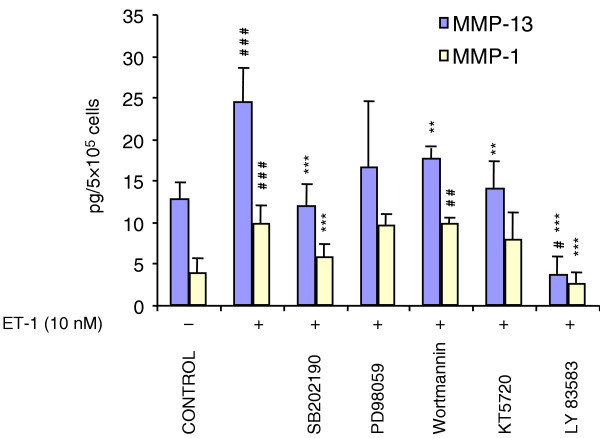
Effect of protein kinase inhibitors and LY83583 on endothelin-1 (ET-1)-induced MMP-13 and MMP-1 production by human osteoarthritis chondrocytes. Confluent monolayer chondrocytes were preincubated 30 min at 37°C with SB 202190 (1 μM), PD98059 (10 μM), Wortmannin (100 nM), KT5720 (4 μM) or LY83583 (2 μM) for 30 min at 37°C, and were then challenged with ET-1 for 24 hours. MMP-13 and MMP-1 proteins were measured in the culture media using specific ELISA assays. *P *values indicate significant differences comparing experimental conditions with ET-1 treatment alone (*) and versus the control cultures (^#^). Values are expressed as the mean ± standard error of the mean of five independent experiments performed in duplicate. Significant differences: ^#, ^* *P *< 0.05; ^##, ^** *P *< 0.01; ^###, ^*** *P *< 0.005.

The effects of ET-1 and those of various inhibitors on MMP-1 production and MMP-13 production are shown in Fig. 1. At 10 nM ET-1 the production of both enzymes was significantly increased (*P *< 0.005). SB202190, a p38 inhibitor, completely suppressed the ET-1-stimulated production of both enzymes, whereas the phosphatidyl inositol 3 kinase inhibitor Wortmannin and the PKA inhibitor KT5720 partially but significantly (*P *< 0.01) decreased the level of MMP-13 only. Interestingly, the most potent inhibitor of MMP-1 and MMP-13 production was LY83583, an inhibitor of NO-dependent soluble guanylate cyclase and of cGMP. This agent not only suppressed the ET-1-induced stimulation, but also decreased the level of both enzymes below the basal level: a significant difference was found for both MMP-13 and MMP-1 when compared with the ET-1 stimulation (*P *< 0.005) and for MMP-13 when compared with the control (*P *< 0.05). Although a decrease in MMP-13 was noted with the MEK1/2 kinase inhibitor PD98059 at the concentration tested, it did not reach statistical significance. With this inhibitor, no effect was found on MMP-1 production.

### ET-1 induces NO production

**Figure 2:**
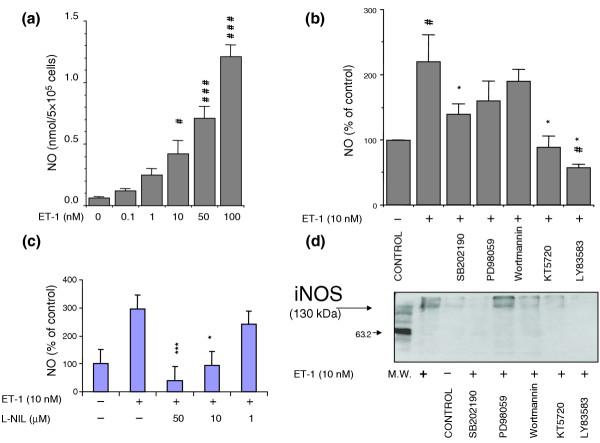
Effect of endothelin-1 (ET-1) on nitric oxide (NO) release and inducible nitric oxide synthase (iNOS) expression by human osteoarthritis (OA) chondrocytes. NO was measured in the culture media, and iNOS protein was detected in cell extracts and revealed by western blot using specific antiserum, as described in Materials and methods. **(a) **Concentration-dependent ET-1-induced NO accumulation in the culture media from confluent human OA chondrocytes treated with ET-1 (0–100 nM) at 37°C for 24 hours. **(b) **Effect of protein kinase inhibitors and of guanylate cyclase inhibitor on ET-1-induced NO release in OA chondrocytes. Confluent monolayer chondrocytes were preincubated with SB 202190 (1 μM), PD98059 (10 μM), Wortmannin (100 nM), KT5720 (4 μM) or LY83583 (2 μM) for 30 min at 37°C and then challenged with ET-1 for 24 hours, and NO was determined in the culture media. **(c) **Effect of iNOS inhibition on NO release induced by ET-1 in human OA chondrocytes. The chondrocytes were pretreated with the allosteric inhibitor of iNOS, L-N^6 ^(1-iminoethyl)lysine (L-NIL) (0–50 μM), for 30 min and were then incubated with ET-1 (10 nM) for an additional 24 hours. The NO level was measured in the culture media. **(d) **Effect of protein kinase inhibitors and LY83583 on ET-1-induced iNOS in human OA chondrocytes. Chondrocytes were preincubated with SB 202190 (1 μM), PD98059 (10 μM), Wortmannin (100 nM), KT5720 (4 μM) or LY83583 (2 μM) for 30 min at 37°C and then challenged with ET-1 for 24 hours, and iNOS was then quantified. M.W., **molecular weight**. (a)–(c) Values are the mean ± standard error of the mean of six independent experiments performed in duplicate. (d) Representative blot of three independent experiments. *P *values indicate the significant difference between ET-1 treated cells and cells treated with indicated inhibitors + ET-1 (*) and versus control (^#^). Significant differences: ^#, ^* *P *< 0.05, ^###, ^*** *P *< 0.005.

The effects of ET-1 on NO release and on iNOS expression are shown in Fig. 2. Figure 2a shows that ET-1 greatly stimulated NO production and was released in a concentration-dependent manner. Incubation with increasing concentrations of ET-1, from 0.1 to 100 nM, augmented almost 12-fold the linear accumulation of NO. To determine the mechanism involved in the ET-1-induced NO production, the effects of the major intracellular signalling pathways were investigated. Figure 2b shows that the ET-1-induced NO release was significantly inhibited by p38 inhibition and prevented by KT5720, a PKA inhibitor. No significant effect was noted for MEK1/2 inhibition by PD98059 and by Wortmannin. Moreover, the guanylate cyclase inhibitor LY83583 reduced the NO production as significant differences were found when compared with either the ET-1 stimulation (*P *< 0.05) or with the control (*P *< 0.05), and this inhibitor also decreased both the endogenous and ET-1-induced iNOS level (Fig. 2d). The ET-1-induced NO release occurs via iNOS as shown in Figure 2c: complete inhibition of iNOS by 50 μM allosteric iNOS inhibitor L-NIL, as expected, almost completely inhibited NO release. Figure 2d shows the effects of various inhibitors on iNOS expression, as determined by western blot analysis of cell extracts. The 24-hour incubation of cells with ET-1 results in an increase of iNOS protein (Fig. 2d, lane 2). The ET-1-induced iNOS protein expression was completely suppressed by SB202190 and LY83583, and was partially suppressed by Wortmannin and KT5720. PD98059 had no effect.

### Intracellular protein kinase phosphorylation in the presence of ET-1

**Figure 3:**
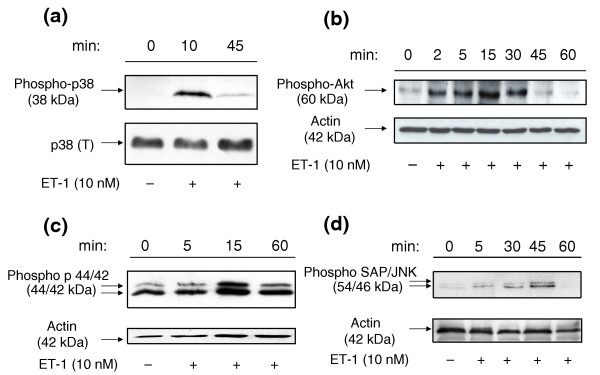
Phosphorylation of p38 mitogen-activated protein (MAP) kinase, Akt, p44/42 and stress-activated protein kinase/Jun-N-terminal kinase (SAP/JNK) by endothelin-1 (ET-1) in human osteoarthritis (OA) chondrocytes. **(a) **Western immunoblot of p38 MAP kinase. Confluent human OA chondrocytes were incubated with ET-1 (10 nM) for 10 or 45 min and the cell extracts were prepared as described in Materials and methods. Western immunoblots used antiserum against activated (phospho-p38) and total p38 MAP kinase (p38 T). Representative result of three different experiments. **(b) **Western immunoblot of Akt. Cells were incubated for 2, 5, 15, 30, 45 or 60 min in the presence of ET-1 (10 nM) and cell extracts were prepared as described in Materials and methods. Western immunoblot was carried out using an antiserum specific for phospho Ser 473 of Akt. Representative result of three different experiments. **(c) **Western immunoblot of p44/42. Confluent human OA chondrocytes were incubated with ET-1 (10 nM) for 0, 5, 15 or 60 min and cell extracts were prepared as described in Materials and methods. **(d) **Western immunoblot of SAP/JNK protein kinase. Confluent human OA chondrocytes were incubated with ET-1 (10 nM) for 0, 5, 30, 45 and 60 min, and cell extracts were prepared as described in Materials and methods. Actin detection was used as a control of the level of proteins loaded. Representative blot of three independent experiments.

Figure 3a–d show the effects of ET-1 on the phosphorylation of p38, Akt, p44/42 and SAP/JNK kinases as detected by western blot of cell extracts. ET-1 at 10 nM induced p38, Akt, p44/42, and SAP/JNK phosphorylation in a time-ordered manner. For p38, the maximal effect following cell exposure to ET-1 was obtained at 10 min. For Akt, the maximal effect was observed at 2 min of cell exposure and this effect persisted during 30 min, followed by a decline at 45 min. At this time (45 min), both p38 kinase and Akt phosphorylated forms were diminished. The maximal effect was obtained at 15 min for p44/42 kinase and at 45 min for SAP/JNK. The SAP/JNK phosphorylated forms were not detected at 60 min, whereas that of p44/42 decreased but was still present even at 60 min.

### ET-1 did not affect apoptosis

As ET-1 induces NO release and because the accumulation of NO causes apoptosis, we explored this potential effect. OA chondrocytes incubated in the absence of (control) or in the presence of ET-1 (10 nM) for 72 hours showed that ET-1 did not affect apoptosis (TUNEL reaction; data not shown) or the production of either anti-apoptotic Bcl2 or pro-apoptotic Bad proteins. A similar percentage of positively stained cells was found for Bcl2 (42.8 ± 5.1% and 43.2 ± 4.3% for the control and for ET-1, respectively) and for Bad (10.1 ± 3.8% and 9.5 ± 2.1%, respectively).

## Discussion

This study shows an overproduction of NO, MMP-1 and MMP-13 in human OA chondrocytes stimulated by ET-1. This result goes beyond previous results [2], which showed that human OA synovial tissue and joint cartilage express the ET-1 gene and overproduce ET-1, resulting in an excessive synthesis of MMP-1 and MMP-13 in the same tissues. In addition, the result goes beyond these findings and enlightens on the mechanism by which ET-1 accomplishes this action. Strong evidence was obtained for the key role played by NO, whose production and release were also upregulated by ET-1.

NO induces smooth muscle cell relaxation by activating soluble guanylate cyclase and by increasing the intracellular concentration of cGMP. LY83583 suppresses the effect of NO by inhibiting this NO-dependent production of cGMP [23]. In the present study, LY83583 was also shown to strongly inhibit MMP-1 and MMP-13 production by unstimulated and ET-1-stimulated OA chondrocytes, showing the key role of cGMP for the synthesis of these enzymes. This finding confirms a previous observation that cGMP is necessary for protein synthesis [9], and brings further evidence that an excess of NO is harmful to cells.

It is generally accepted that progressive tissue destruction in rheumatoid arthritis and in OA results from an excessive breakdown mediated by various proteolytic enzymes and other catabolic agents such as free radicals and NO [1,13,24,25]. Our results suggest that ET-1 should also be added to the list of potential deleterious agents that may account for articular cartilage destruction in rheumatic diseases. The action of ET-1 seems to be dual via an increase in MMP and NO production. ET-1-induced stimulation of MMP-1 and MMP-13, as well as the induction of iNOS gene expression with subsequent NO overproduction by OA chondrocytes, may interfere with the proinflammatory cytokine pathways. Indeed, we and other workers have shown that IL-1β upregulates the synthesis of ET-1 [3], which in turn can induce IL-1β gene transcription and consequently the production of the protein [26]. We previously demonstrated [2] that MMP-13 expression was induced similarly by ET-1 and IL-1β; however, although they both enhanced MMP-1 expression, the effect of IL-1β was more potent on this enzyme. Interestingly, using a specific immunoassay measuring the C telopeptide of type II collagen fragments on OA cartilage explants, we also found that the level of the cleaved collagen fragments were significantly increased in the presence of both IL-1β and ET-1 with a more potent effect observed for ET-1. This could be explained by a putative synergy between ET-1 and IL-1β as ET-1 induces IL-1β and as IL-1β has a positive feedback on ET-1 synthesis [19,27].

NO is an important signalling molecule at physiological concentrations [28], but when overproduced via iNOS gene activation it is toxic to cells [29]. NO triggers the transcription of several proinflammatory genes [28,30], interacts with the cysteine residues of many proteins (S nitrosylation) and may alter their structure and function. In the presence of the superoxide anion, NO generates peroxynitrite and hydroxyl radicals that are cytotoxic, inducing peroxidation of lipids and damaging other molecules, such as DNA, and matrix macromolecules. This finally results in the inhibition of many cellular processes that impair the capacity of the cells to synthesize matrix macromolecules and to repair damaged tissue [8,31].

In addition to the findings already discussed, the present study sheds more light on the major signalling pathways involved in the ET-1-induced MMP-1 and MMP-13 production and in NO production. In OA chondrocytes, ET-1 seems to stimulate the production of these enzymes through activation of, at least, two kinases, p38 MAP kinase and PKA. As shown by western blot analysis of the cell extracts, incubation of cells for a short period of time with ET-1 results in the phosphorylation of p38 MAP, p44/42, SAP/JNK and Akt kinases. This effect occurs within minutes following a challenge with ET-1, and disappears after 45 and 60 min for the p-38 and SAP/JNK kinases, respectively. The activation of these kinases is probably necessary for the induction by ET-1 of MMP-1 production and MMP-13 production. The inhibition of p38 kinase is associated with a suppression of the ET-1-induced stimulation of both enzymes, whereas the inhibitions of adenyl cyclase-dependent PKA kinase is associated with a partial suppression of the ET-1-induced stimulation of MMP-13 production only. This suggests that these inhibitors are specific for the ET-1-activated pathways since they do not affect the basal levels of MMP-1 and MMP-13.

Another point also deserves consideration. Tardif and colleagues [32] have described two OA chondrocyte populations distinctive by their MMP-13 content and their response to IL-1β. One population contains small amounts of MMP-13 protein and is highly sensitive to IL-1β stimulation; the other population is enriched in MMP-13 protein but poorly responds to the cytokine. The cell heterogeneity of OA cartilage may explain some variability of the results observed in our study, particularly in the case of using low doses of the MEK1/2 inhibition followed by ET-1 stimulation. In fact, when MAP kinase pathways (extracellular signal-regulated kinase, JNK and p38) are activated in chondrocytes, their inhibition is dependent of the inhibitor concentration used, particularly for SB 203580 and PD 98059 [18]. PD 98059, which had no effect in the present study at the concentration of 10 μM on ET-1-induced iNOS expression and NO production, was demonstrated in other studies to suppress NO induction in human chondrocytes, as shown by Gemba and colleagues [18].

The phosphorylation of p38 MAP kinase by ET-1 was also described in osteoblast-like cells [33] and in cardiac myocytes [34], while in chondrocytes overproducing MMP-1 and MMP-13 this MAP kinase was shown to be phosphorylated principally by IL-1β [35]. Activation of PKA was shown to be required for the upregulation of iNOS, and for the subsequent production and release of NO by several cell types such as vascular smooth muscle cells [36], cardiac myocytes [37] and human macrophages [38]. It is also associated with the cytokine-induced NO production in human OA articular chondrocytes [39]. Our results suggest that the activation of PKA is also required for the ET-1-induced upregulation of iNOS and for subsequent production of NO by human OA chondrocytes. However, PKA activation seems to be less required for the ET-1-induced upregulation of MMP-13 and not at all necessary for the upregulation of MMP-1 since the inhibition of PKA with KT5720 does not affect the ET-1-induced overproduction of this enzyme. In the present study, subtle differences are shown in the pattern of inhibition of the ET-1-induced overproduction of MMP-1 and MMP-13. The effect of ET-1 on MMP-13 production was more sensitive to the inhibitors of protein kinases than on MMP-1 production. As suggested earlier, these variable responses point to possible different cell populations producing these two enzymes or to relevant signalling pathways eliciting the ET-1-induced stimulations [35].

We also tested the hypothesis that ET-1 may act in OA through induction of apoptosis. This was based on the findings that cells of the superficial layer disappear during cartilage degeneration [40], that ET-1 is preferentially produced in this layer [2], and that NO may induce apoptosis and cell death at high concentrations [29]. Indeed, chondrocyte death may represent one of the contributing factors in cartilage destruction. However, as shown in the present study, ET-1 does not appear to induce chondrocyte apoptosis or cell death. Using the TUNEL technique (which was recently shown to detect both apoptosis and cell death [29]), and using Bcl2 and Bad protein determination, no differences were found between ET-1-treated cultures and control cultures.

## Conclusion

The present study shows that ET-1 causes an overproduction of NO, MMP-1 and MMP-13 in human OA chondrocytes. The signalling pathway used by ET-1 in these cells was also demonstrated. The fact that ET-1 possesses the biological properties described acknowledges this peptide as an important catabolic factor contributing to the cartilage destruction via induction of the deleterious molecules such as MMPs and NO. NO seems to be a key molecule that is produced in parallel with the ET-1-induced overproduction of the MMPs. Blocking the effects of ET-1 may thus become a useful therapeutic approach aimed at stopping cartilage destruction in rheumatic conditions such as rheumatoid arthritis and OA.

## Abbreviations

DMEM = Dulbecco's modified Eagle's medium; ELISA = enzyme-linked immunosorbent assay; ET-1 = endothelin-1; FCS = foetal calf serum; IL = interleukin; iNOS = inducible nitric oxide synthase; L-NIL = L-N^6^(1-iminoethyl)lysine; MAP = mitogen-activated protein; MEK1/2 = mitogen-activated protein kinase kinase 1/2; MMP = metalloprotease; NO = nitric oxide; OA = osteoarthritis; PKA = protein kinase A; SAP/JNK = stress-activated protein kinase/Jun-N-terminal kinase; TUNEL = terminal deoxynucleotidyl transferase-medulated dUTP nick end labelling.

## Competing interests

The author(s) declare there are no competing interests.

## Authors' contributions

CAM executed the study, contributed to the preparation of the manuscript and performed statistical analysis. MR-B and FSS assisted in the experiments and in the isolation of chondrocytes from human cartilage. JCF, JM-P and J-PP assisted with the design of experiments and obtained human tissues. DRM evaluated and interpreted data and assisted with the preparation of the manuscript. FM designed the study, supervised the project, evaluated and interpreted data, and prepared the manuscript.
